# Extensive Nosocomial Transmission of Measles Originating in Cruise Ship Passenger, Sardinia, Italy, 2014

**DOI:** 10.3201/eid2108.141105

**Published:** 2015-08

**Authors:** Antonietta Filia, Antonino Bella, Giovanna Cadeddu, Maria Rafaela Milia, Martina Del Manso, Maria Cristina Rota, Fabio Magurano, Loredana Nicoletti, Silvia Declich

**Affiliations:** Istituto Superiore di Sanità, Rome, Italy (A. Filia, A. Bella, M. Del Manso, M.C. Rota, F. Magurano, L. Nicoletti, S. Declich);; Local Health Unit 7, Carbonia, Sardinia, Italy (G. Cadeddu, M.R. Milia)

**Keywords:** measles, measles virus, viruses, outbreak, nosocomial transmission, infection control, cruise ship passenger, Sardinia, Italy

## Abstract

We report a measles outbreak in Sardinia, Italy, that originated in a cruise ship passenger. The outbreak showed extensive nosocomial transmission (44 of 80 cases). To minimize nosocomial transmission, health care facilities should ensure that susceptible health care workers are vaccinated against measles and should implement effective infection control procedures.

Measles is a highly infectious acute viral disease that can cause severe complications. It is transmitted by direct contact with large respiratory droplets and by the airborne route. This disease will develop in ≈90% of susceptible close contacts of infectious persons. We report a large outbreak of measles in Sardinia, Italy, that originated from a reported outbreak on a cruise ship (*1*) and showed extensive nosocomial transmission.

## The Study

A rash developed in the index case-patient (an unvaccinated woman) on February 23, 2014, nine days after she disembarked the cruise ship on which she had traveled during February 6–14, and returned to Sardinia. While infectious, she was seen in the emergency department (ED) of a local hospital, where she was admitted for 3 days for laboratory-confirmed measles. The index case-patient transmitted the infection to 6 persons: a coworker (onset of rash March 6), 1 adult relative (onset of rash March 10), 1 adult visitor to the ED in which the index case-patient sought medical care (onset of rash March 11), and 3 health care workers (HCWs) in the same hospital (onset of rash March 2, 10, and 11). 

Transmission continued in families, work settings, and the hospital setting. As of July 2014, a total of 136 cases from the cruise ship outbreak were reported to the national measles surveillance system in Italy, of which 80 were from the secondary outbreak described in this report (Figure). The remaining 56 cases include 28 primary cases (crew and passengers) and 28 secondary cases; 39 of 56 case-patients were Italian and 17 were of foreign origin. One other secondary outbreak linked to the cruise ship has been reported (*2*).

**Figure:**
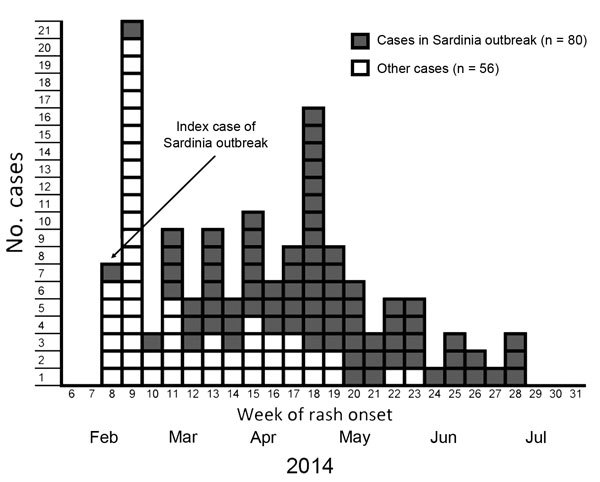
Number of primary and secondary cases (n = 136), by week of rash onset, during a measles outbreak that originated in a cruise ship passenger, including cases reported in a secondary outbreak, Sardinia, Italy, February–July 2014.

Classification of measles cases was based on European Union 2012 case definitions (*3*). A probable case-patient was any person meeting clinical criteria for measles and having an epidemiologic link to the outbreak who did not undergo laboratory testing. A confirmed case-patient was any person with an epidemiological link to the outbreak meeting clinical criteria and having laboratory evidence of infection (positive IgM serologic result or identification of measles virus RNA by PCR). A nosocomial case-patient was defined as any person with measles who had contact with a probable or confirmed case-patient in the hospital (including the waiting area of the ED) 7–21 days before rash onset and had no other source identified. An HCW was any hospital staff or other worker who had regular contact with patients, including clinicians, nurses, students in these disciplines, paramedical professionals, social workers, ambulance workers, porters, and other hospital support staff.

Median age of 80 case-patients was 26 years (range 8 months–55 years); 50 (62.5%) were female. Vaccination status was known for 76/80 (95.0%) patients, of whom 74 (97.4%) were unvaccinated and 2 (2.6%). had received only 1 dose of measles vaccine. Forty-one (51.3%) patients reported >1 complication, and 19 (23.8%) reported >2 complications (Table). Thirty-five (44.9%) of 78 patients were hospitalized, including 2 patients admitted to the intensive care unit for respiratory insufficiency. In addition, 14 patients were seen in the ED.

**Table:** Complications in 80 case-patients during a measles outbreak linked to a cruise ship passenger, Sardinia, Italy, February–July 2014

**Complication**	**No. (%) case-patients**
**Diarrhea**	22 (27.5)
**Keratoconjunctivitis**	9 (11.3)
Hepatitis*	8 (10.0)
**Otitis media**	7 (8.8)
**Pneumonia**	7 (8.8)
**Stomatitis**	7 (8.8)
**Laryngotracheobronchitis**	3 (3.8)
**Respiratory insufficiency**	2 (2.5)
**Other**	2 (2.5)

*Increased levels of aminotransferases but no jaundice.

Thirty-four cases (42.5%) were laboratory confirmed; the remaining 46 cases were classified as probable. Measles virus genotype B3 was identified in 7 cases, and phylogenetic analysis showed that viral sequences were identical to each other and to those obtained from the cruise ship outbreak (*2**,**4*) (measles nucleotide surveillance [MeaNS] database accession nos. 62563, 62564, 67009, 67016, 62565, 67017, and 67020). Nosocomial cases included HCWs (n = 15; 18.8%) and persons infected in the waiting area of the ED or in a hospital ward (n = 29; 36.2%). Six additional case-patients were infected by an HCW in a family setting.

Local health authorities conducted case investigations, including active case finding by regular visits/phone calls to the hospital, and contact tracing and vaccination of susceptible contacts. Hospital directors were contacted by local health authorities to inform them of measles transmission in the hospital. No data were available for vaccination coverage among HCWs because this information is not routinely obtained. An information circular that invited susceptible staff to receive measles vaccine and consent/dissent forms were distributed in the affected wards (ED, intensive care unit, medicine). However, participation has been negligible: only 2 of 114 HCWs agreed to be vaccinated.

## Conclusions

Clinical and genotype data suggest that the index case-patient for this outbreak was infected during the cruise, either onboard or in 1 of the cities visited during the cruise (*5*). The index case-patient triggered an outbreak with extensive spread, which highlights the ease with which measles is transmitted in various settings, especially the hospital setting, which accounted for 44 (55%) of the cases.

Nosocomial transmission of measles has been frequently reported and has a major role in the epidemiology of this disease, especially in industrialized countries (*6**,**7*). As described in this outbreak, nosocomial transmission can lead to measles in HCWs, other patients at the hospital, and susceptible hospital visitors. Measles is one of the most contagious viral diseases and patients frequently visit hospitals or health care facilities for diagnosis and management, which might lead to nosocomial transmission if appropriate infection control measures are not immediately instituted (*8*).

The potential for airborne transmission of measles in waiting areas of health care facilities is higher than for other airborne infections, such as tuberculosis and influenza, and persons might become infected after a relatively short exposure time (*9*). Also, measles virus can survive for up to 2 hours in the air or on objects and surfaces, which indicates that a susceptible person can be infected even after an infected person has left the area (*10**,**11*). Infectivity of measles is greatest during the 3 days before the onset of rash, when the disease might not be suspected (*12*).

The consequences of measles transmission in a health care setting are serious because the infection might be transmitted to immunocompromised patients, young children, pregnant women, or other persons at high risk for severe complications. More than 50% of the case-patients in the described outbreak reported complications, and respiratory insufficiency developed in 2 patients. In addition to illness and death attributable to measles, nosocomial transmission also leads to major use of resources for evaluating and containing an outbreak (*11*).

Measles is effectively prevented by a 2-dose vaccination, and a key preventative measure against nosocomial transmission is vaccination of HCWs (*13*). In Italy, measles vaccine is recommended for all susceptible HCWs. However, HCWs are not required to show evidence of measles immunity for employment. Analysis of national measles surveillance data for Italy during October 2010–December 2011 showed that HCWs accounted for 11.6% of reported case-patients for whom information on occupation was recorded (*14*).

In addition to maintaining high coverage of vaccination in the community, ensuring that susceptible HCWs are vaccinated against measles, and maintaining a complete staff immunity database, it is essential that isolation protocols and infection control guidelines be in place in hospitals to minimize nosocomial spread of infection. Infection control measures should include maintaining a high level of awareness among staff, excluding exposed susceptible HCWs from work during the measles incubation period; immediately isolating suspected case-patients who come to an ED or any outpatient waiting area, contacting persons known to be exposed in hospital or outpatient (including ED) settings, offering postexposure vaccination to susceptible persons, and strengthening surveillance for nosocomially acquired cases (*7**,**10**,**11**,**15*). Stronger recommendations and guidelines are needed at the national level and in Europe.
